# Multi-Locus Genome-Wide Association Study and Genomic Selection of Kernel Moisture Content at the Harvest Stage in Maize

**DOI:** 10.3389/fpls.2021.697688

**Published:** 2021-07-09

**Authors:** Guangfei Zhou, Qiuli Zhu, Yuxiang Mao, Guoqing Chen, Lin Xue, Huhua Lu, Mingliang Shi, Zhenliang Zhang, Xudong Song, Huimin Zhang, Derong Hao

**Affiliations:** ^1^Department of Food Crops, Jiangsu Yanjiang Institute of Agricultural Science, Nantong, China; ^2^Jiangsu Collaborative Innovation Centre for Modern Crop Production, Nanjing, China; ^3^Jiangsu Nantong Crop Cultivation Technique Direction Station, Nantong, China

**Keywords:** maize (*Zea mays* L), kernel moisture content, multi-locus genome-wide association study, quantitative trait nucleotide, candidate gene, genomic selection

## Abstract

Kernel moisture content at the harvest stage (KMC) is an important trait that affects the mechanical harvesting of maize grain, and the identification of genetic loci for KMC is beneficial for maize molecular breeding. In this study, we performed a multi-locus genome-wide association study (ML-GWAS) to identify quantitative trait nucleotides (QTNs) for KMC using an association mapping panel of 251 maize inbred lines that were genotyped with an Affymetrix CGMB56K SNP Array and phenotypically evaluated in three environments. Ninety-eight QTNs for KMC were detected using six ML-GWAS models (mrMLM, FASTmrMLM, FASTmrEMMA, PLARmEB, PKWmEB, and ISIS EM-BLASSO). Eleven of these QTNs were considered to be stable, as they were detected by at least four ML-GWAS models under a uniformed environment or in at least two environments and BLUP using the same ML-GWAS model. With *qKMC5.6* removed, the remaining 10 stable QTNs explained <10% of the phenotypic variation, suggesting that KMC is mainly controlled by multiple minor-effect genetic loci. A total of 63 candidate genes were predicted from the 11 stable QTNs, and 10 candidate genes were highly expressed in the kernel at different time points after pollination. High prediction accuracy was achieved when the KMC-associated QTNs were included as fixed effects in genomic selection, and the best strategy was to integrate all KMC QTNs identified by all six ML-GWAS models. These results further our understanding of the genetic architecture of KMC and highlight the potential of genomic selection for KMC in maize breeding.

## Introduction

Kernel moisture content at the harvest stage (KMC) is one of the important traits that influence maize mechanical harvesting, especially in high latitude areas (Sala et al., 2012; Li et al., 2017). Since the 1970s, many developed countries, such as the United States and Germany, have achieved fully mechanical harvesting of maize. By contrast, other countries, like China, have not yet implemented mechanical harvesting, primarily due to a lack of suitable maize varieties (Liu et al., 2013). The high KMC of currently used maize varieties restricts mechanical harvesting and represents the major barrier to maize development in China (Zhou et al., 2016, 2020; Li et al., 2017). Therefore, the genetic improvement of KMC and the breeding of elite varieties with low KMC is a major goal for maize breeders in China.

The loss of maize kernel moisture occurs in two phases. The first phase lasts from pollination to kernel physiological maturity. During this phase, water in the kernel is replaced with carbohydrates, oils, proteins, etc., and the moisture of the kernel is highly dependent on its own physiological characteristics; this is called the physiological dehydration stage. The second phase lasts from physiological maturity to harvest. During this phase, the change in kernel moisture is primarily caused by moisture evaporation into the air and is thus readily influenced by environmental factors and other agronomic traits; this is known as the field dehydration stage (Brooking, 1990; Reid et al., 2010). Rapid rates of kernel filling and field dehydration are marked features of maize varieties with low KMC (Johnson and Tanner, 1972; Sala et al., 2006). Fewer husk layers as well as shorter and lighter husks are associated with greater loss of kernel moisture after physiological maturity (Reid et al., 2010; Li et al., 2016; Zhou et al., 2018). In addition, moisture from the kernels can be transported to other plant parts through the cob and stem in response to water potential differences (Zhou et al., 2018).

Previous studies have revealed that KMC is controlled by numerous quantitative trait loci (QTLs), and hundreds of QTLs for maize KMC have been identified (Beavis et al., 1994; Melchinger et al., 1998; Austin et al., 2000; Ho et al., 2002; Mihaljevic et al., 2004, 2005; Blanc et al., 2006; Sala et al., 2006; Frascaroli et al., 2007; Capelle et al., 2010; Kebede et al., 2016; Song et al., 2017; Zhou et al., 2018; Liu et al., 2020; Yin et al., 2020b; Zhang et al., 2020; Li et al., 2021). Using meta-analysis, 44 and 34 meta-QTLs for KMC were identified by Xiang et al. (2012) and Sala et al. (2012), respectively. Liu et al. (2020) narrowed a major QTL for KMC (*qGwc1.1*) to a 2.05-Mb genomic region on chromosome 1 using a recombinant-derived progeny test. Li et al. (2021) cloned a gene (gar2-related nucleolar protein, *GAR2*) for KMC on maize chromosome 7. Yin et al. (2020b) identified seven QTLs for KMC using multiple-environment analysis and revealed that the interactions between QTLs and the environment were larger than their additive effects. Zhou et al. (2018) detected five QTLs for KMC through a mixed linear model (MLM) of single-locus genome-wide association study (SL-GWAS).

The Bonferroni correction for multiple tests is frequently used in SL-GWAS to reduce spurious associations, and this results in the elimination of some positive loci with small effects. Multi-locus GWAS (ML-GWAS), an alternative GWAS method, was developed to address this issue; it considers the information from all markers simultaneously and does not require a multiple testing correction. ML-GWAS has been shown to have higher power and accuracy for the detection of quantitative trait nucleotides (QTNs) in maize. Zhang et al. (2018) used four ML-GWAS methods (mrMLM, FASTmrEMMA, ISIS EM-BLASSO, and pLARmEB) to identify QTNs for three stalk lodging resistance-related traits in maize and reported that the methods were reliable and complementary. Xu et al. (2018) compared one SL-GWAS method (GEMMA) and three ML-GWAS methods (FASTmrEMMA, FarmCPU, and LASSO) for the genetic detection of maize starch pasting properties, and more QTNs were detected by individual ML-GWAS methods than by the SL-GWAS method. An et al. (2020) used one SL-GWAS method (MLM) and six ML-GWAS methods (mrMLM, FASTmrMLM, FASTmrEMMA, pLARmEB, pKWmEB, and ISIS EMBLASSO) to dissect the genetic architecture of maize kernel row number. The largest number of QTNs were identified with the mrMLM method, and the most co-detected QTNs were identified with ISIS EM-BLASSO.

Given the lack of large-effect QTLs, the use of marker-assisted selection (MAS) for KMC is not ideal in maize breeding programs, and it is necessary to incorporate far more markers. Genomic selection (GS), an upgraded form of MAS, aims to use genetic effects of genome-wide molecular markers to estimate the genomic estimated breeding value (GEBV) of individuals based on optimum statistical models (Meuwissen et al., 2001). This approach has been considered most promising for the genetic improvement of complex traits controlled by multiple genes with minor effects (Wang X. et al., 2018; Xu et al., 2020). Controlling costs by using fewer markers while still achieving accurate predictions for complex quantitative traits remains a challenge (Xu et al., 2020). Recently, several studies reported that taking association markers for interesting traits detected by GWAS into account and including them as fixed effects in GS models resulted in higher accuracy than that achieved with GS models using genome-wide markers (Spindel et al., 2016; Qin et al., 2019; Ravelombola et al., 2019; An et al., 2020; Sehgal et al., 2020).

In this study, we used 251 maize inbred lines that were genotyped using an Affymetrix CGMB56K SNP Array and phenotypically evaluated in three field trials to (i) identify significant QTNs for KMC using ML-GWAS, (ii) predict candidate genes associated with KMC, and (iii) explore the potential of GS for KMC in maize.

## Materials and Methods

### Plant Material and Field Experiments

An association mapping panel of 251 diverse maize inbred lines was used as the plant material in this study (Supplementary Table 1).

The field experiments were performed in three environments in 2020: Nantong, Jiangsu Province (NT, 120°E, 31°N), which is in mid-eastern China and has an average temperature of 15.1°C and an average rainfall of 1,040 mm per year; Xinxiang, Henan Province (XX, 113°E, 35°N), which is in the middle of China and has an average temperature of 15.5°C and an average rainfall of 573.4 mm per year; and Sanya, Hainan Province (SY, 108°E, 18°N), which is in southern China and has an average temperature of 25.7°C and an average rainfall of 1,347 mm per year. Each line was grown in single rows, which were 3 m in length and spaced 0.6 m apart, thereby giving a planting density of 65,000 plants/ha. The trial followed a randomized complete block design with two replicates per environment. The agronomic management of the field experiments was the same in the three environments.

### Phenotypic Evaluation and Data Analysis

As described in our previous study (Zhou et al., 2018), before the experimental treatment, the physiological maturity of each line was evaluated in the field. According to their growth periods, the 251 maize inbred lines were sown on three separate dates to obtain similar physiological maturity in each environment (Supplementary Table 1). In NT, they were planted on the 22nd, 26th, and 30th of March, and the harvest stage was adjusted to between the 24th and 26th of July. In XX, they were planted on the 1st, 5th, and 9th of June, and the harvest stage was adjusted to between the 26th and 28th of September. In SY, they were planted on the 14th, 18th, and 22nd of November, and the harvest stage was adjusted to between the 5th and 7th of the following March. The ears were bagged before silking, and artificial pollination was performed at the same time for each line. The KMC for 6–8 uniformly growing plants in the middle of rows was measured using a hand-held moisture meter. At 10 days after physiological maturity, the KMC of each plant was recorded one time at the middle part of the ear.

The phenotypic data were analyzed using R version 3.6.3 for Windows (https://www.r-project.org/). Analysis of variance (ANOVA) of KMC was performed using *lmer* function of the lme4 package based on the following model: *y*_*ijk*_ = μ + *G*_*i*_ + *E*_*j*_ + *GE*_*ij*_ + *R*_*jk*_ + *e*_*ijk*_, where *y*_*ijk*_ is the KMC on the *i*^th^ genotype in the *j*^th^ environment and *k*^th^ replication, μ is the grand mean over all environments, *G*_*i*_ is the genotypic effect of the *i*^th^ genotype, *E*_*j*_ is the environmental effect of the *j*^th^ environment, *GE*_ij_ is the genotype × environment interaction effect of the *i*^th^ genotype and *j*^th^ environment, *R*_*jk*_ is the effect of the *k*^th^ replication in *j*^th^ environment, and *e*_*ijk*_ is the residual error. The trait heritability (*H*^2^) was estimated following Knapp et al. (1985): the individual environment *H*^2^ (%) = $\sigma_{\text{g}}^{2}$/($\sigma_{\text{g}}^{2}$ $+\sigma_{\text{e}}^{2}$/*r*) × 100%, and the multiple environments *H*^2^ (%) = $\sigma_{\text{g}}^{2}$/($\sigma_{\text{g}}^{2}+\sigma_{\text{ge}}^{2}$/*n*$+\sigma_{\text{e}}^{2}$/*nr*) × 100%, where $\sigma_{\text{g}}^{2}$ is the genotypic variance, $\sigma_{\text{ge}}^{2}$ is the variance for the interaction of genotype with environment, $\sigma_{\text{e}}^{2}$ is the error variance, *n* is the number of environments, and *r* is the number of replications. To minimize the effects of the environment, the best linear unbiased prediction (BLUP) for KMC across the three environments was estimated using the *lmer* function of the lme4 package with the same ANOVA model. The normal distribution test (W-value) of KMC in each environment was estimated using the *Shapiro.test* function of the stats package.

### Genotyping, Population Structure, Linkage Disequilibrium, and Relative Kinship

Genotypes of the 251 maize inbred lines were evaluated using an Affymetrix CGMB56K SNP Array, which contains 56,000 single nucleotide polymorphisms (SNPs) and is made by China Golden Marker (Beijing) Biotech Co., Beijing, China. After quality control, 32,853 SNPs with minor allele frequencies >5% and missing data <20% were used for subsequent analysis. The genotypic data can be downloaded from the website https://pan.baidu.com/s/1_V0fm7hsxNdMbyYzciQsWg.

Population structure was assessed using STRUCTURE 2.3 (Pritchard et al., 2000). The number of subpopulations (*K*) was set from 1 to 10 with five independent runs for each *K*. Both burn-in periods and Markov chain Monte Carlo replication number were set at 100,000 in each run under the admixture model. The *K* value was estimated by the log likelihood of the data [LnP(D)] and an *ad hoc* statistic ΔK, based on the rate of change of LnP(D) between successive *K* values (Evanno et al., 2005). Nei's genetic distance (Nei, 1972) was calculated and used to construct a neighbor-joining tree with MEGA-X software (Kumar et al., 2018). The linkage disequilibrium (LD) parameter *r*^2^ between pairwise SNPs was calculated with PLINK (Purcell et al., 2007), which window size was set at 1,000 kb and *r*^2^ was set at 0.2. The relative kinship matrix of the 251 lines was computed using SPAGeDi 1.3 (Hardy and Vekemans, 2002) with negative values between two individuals set to zero.

### Multi-Locus Genome-Wide Association Study

ML-GWAS was conducted using the mrMLM package (https://cran.r-project.org/web/packages/mrMLM/index.html), including six statistical models: mrMLM (Wang et al., 2016), FASTmrMLM (Tamba and Zhang, 2018), FASTmrEMMA (Wen et al., 2018), pLARmEB (Zhang et al., 2017), pKWmEB (Ren et al., 2018), and ISIS EM-BLASSO (Tamba et al., 2017). The mrMLM is a multi-locus model including markers selected from the random-SNP-effect MLM with a less stringent selection criterion (Wang et al., 2016). The FASTmrMLM is relatively faster with higher statistical power and accuracy in estimating QTNs as compared to mrMLM (Tamba and Zhang, 2018). The FASTmrEMMA combines the MLM and the expectation maximization empirical Bayes method. The pLARmEB integrates least angle regression with empirical Bayes (Zhang et al., 2017), while the pKWmEB integrates Kruskal-Wallis test with empirical Bayes (Ren et al., 2018). The ISIS EMBLASSO can detect significant associations with highest robustness and accuracy as compared to mrMLM and FASTmrEMMA (Tamba et al., 2017). Default values were used for all parameters, and the threshold of logarithm of odds (LOD) ≥ 3 (or *P* ≤ 0.0002) was selected to determine significant QTNs (Zhang et al., 2019). To confirm the efficiency of ML-GWAS, one widely used SL-GWAS method, MLM, was conducted using TASSEL 5.0 (Bradbury et al., 2007), controlling for population structure and kinship. The threshold was also set at *P* ≤ 0.0002 [-log_10_(*P*) ≥ 3.70].

Two types of QTNs were defined as stable QTNs. One is model-stable QTN (msQTN), which is identified by at least four ML-GWAS models under a uniformed environment, another is environment-stable QTN (esQTN), which is identified by in at least two environments and BLUP using the same ML-GWAS model.

### Candidate Gene Analysis

Based on the B73 reference genome v4 (https://www.maizegdb.org/gbrowse), the available genes within regions from 100 kb upstream to 100 kb downstream (LD of the association mapping panel) around the stable QTNs were regards as candidates. Candidate gene annotation was performed at NCBI (https://www.ncbi.nlm.nih.gov/). Expression data for candidate genes were collected from qTeller (https://qteller.maizegdb.org/).

### Genomic Selection

GS was performed using the ridge regression best linear unbiased predictor (rrBLUP) model (Meuwissen et al., 2001) in rrBLUP package of R software (Endelman, 2011). The rrBLUP model (model 1) was: $y_{i}=\mu +\overset{p}{\underset{k=1}{\sum }}x_{ik}\beta_{k}+\varepsilon_{i}$, where *y*_*i*_ is the predicted phenotype of *i*^th^ individual, μ is the grand mean, *x*_*ik*_ is the genotype at the *k*^th^ marker of the *i*^th^ individual, *p* is the total number of markers (i.e., 32,853 high quality markers from the Affymetrix array), β_*k*_ is the estimated random additive marker effect of the *k*^th^ marker, and ε_*i*_ is the residual error. To improve the prediction accuracy, the significant markers identified by ML-GWAS were included as fixed effects in the following model (model 2): $y_{i}=\mu +\overset{m}{\underset{j=1}{\sum }}x_{ik}\alpha_{j}+\overset{p}{\underset{k=1}{\sum }}x_{ik}\beta_{k}+\varepsilon_{i}$, where *m* is the number of significant markers identified ML-GWAS considered as fixed effect covariates, α_*j*_ is the fixed additive effect of the *j*^th^ marker, and the remaining terms are the same as those described in model 1. Twelve sets of markers were included as fixed effects in model 2: the significant markers identified by mrMLM, FASTmrMLM, FASTmrEMMA, pLARmEB, pKWmEB, and ISIS EM-BLASSO; the markers identified by at least two, three, four, five, or six models; and all significant markers identified by all six models.

Prediction accuracy was evaluated using 5-fold cross validation with 100 iterations. In brief, the association panel was randomly divided into five equal subsets; four subsets were regarded as the training population, and the remaining set was considered to be the testing population. The prediction accuracy was defined as the coefficient of determination between the predicted and the observed values based on the linear regression analysis.

## Results

### Population Structure, Linkage Disequilibrium, and Relative Kinship

**Figure 1:**
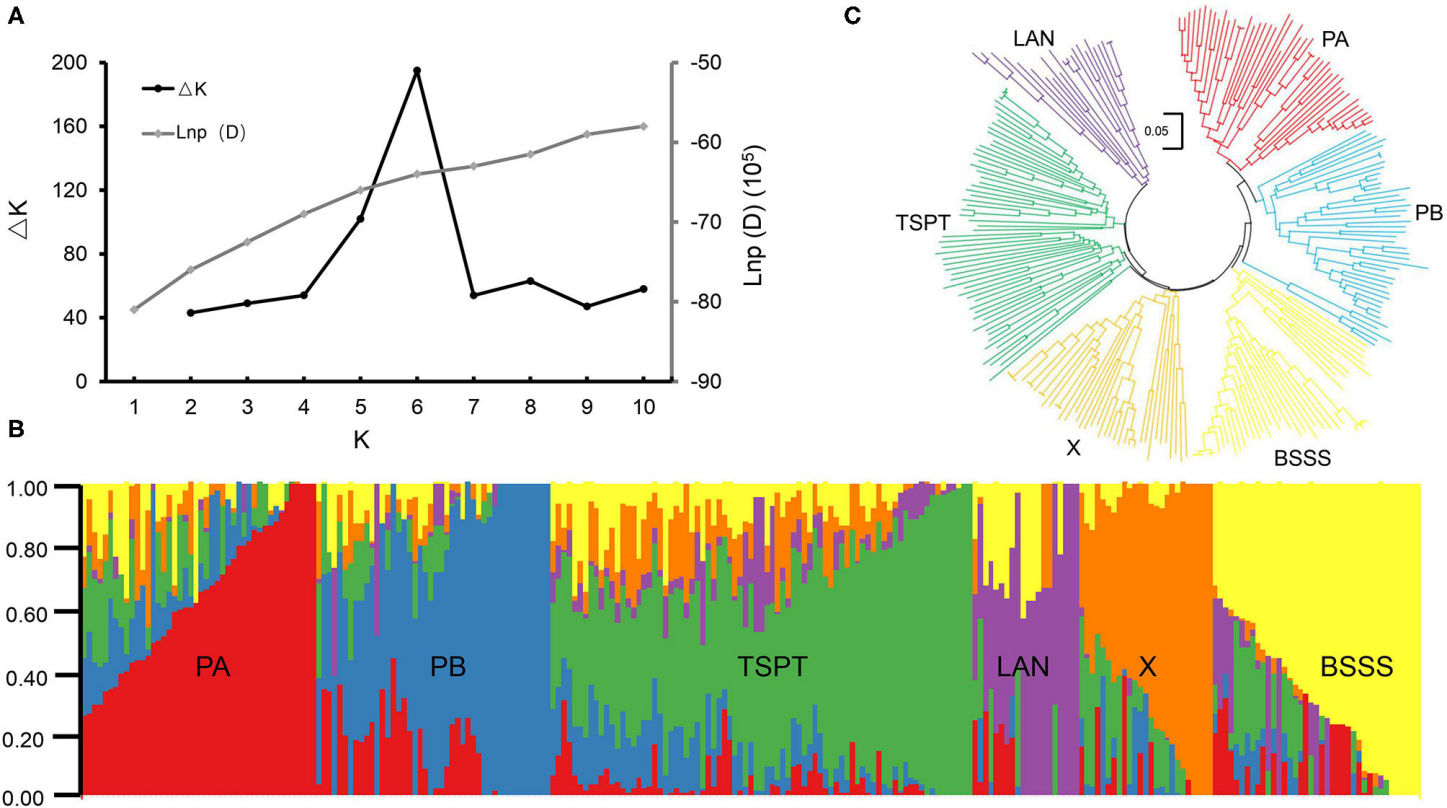
Population structure analysis of 251 maize inbred lines. **(A)** Estimated ΔK and LnP(D) in the STRUCUTRE analysis. **(B)** Neighbor-joining tree of 251 maize inbred lines. **(C)** The Bayes cluster plot of 251 maize inbred lines when K = 6.

STRUCTURE software was used to calculate the Bayesian clustering from *K* = 1 to 10 with five independent runs for each *K*. The LnP(D) values increased as *K* increased from 1 to 10 without an obvious inflection point, and ΔK reached its peak at *K* = 6 (Figure 1A), suggesting that this association mapping panel could be divided into six subgroups (Figure 1B). A neighbor-joining tree was constructed based on Nei's genetic distance, and it showed six main clusters for this panel (Figure 1C), consistent with the STRUCTURE results.

The six subpopulations were designated PA, PB, Tangsipingtou (TSPT), Lancaster (LAN), BSSS, and X (Supplementary Table 1). Subgroup PA, including 45 inbred lines, tended to be improved Reid lines, such as Zheng 58 and Ye478. Subgroup PB, including 45 inbred lines, derived mainly from hybrid 78599 and included Qi319, ND1145, etc. Subgroup TSPT, including 74 inbred lines, consisted mainly of inbred lines such as Huangzaosi and Chang7-2. Subgroup LAN, including 15 inbred lines, contained the representative inbred line Mo17. Subgroup BSSS derived from the synthetic variety BSSS and included 44 inbred lines; its representative inbred line was B73. Subgroup X, including 28 inbred lines, derived mainly from hybrid Xianyu335 that is widely cultivated in China.

**Figure 2:**
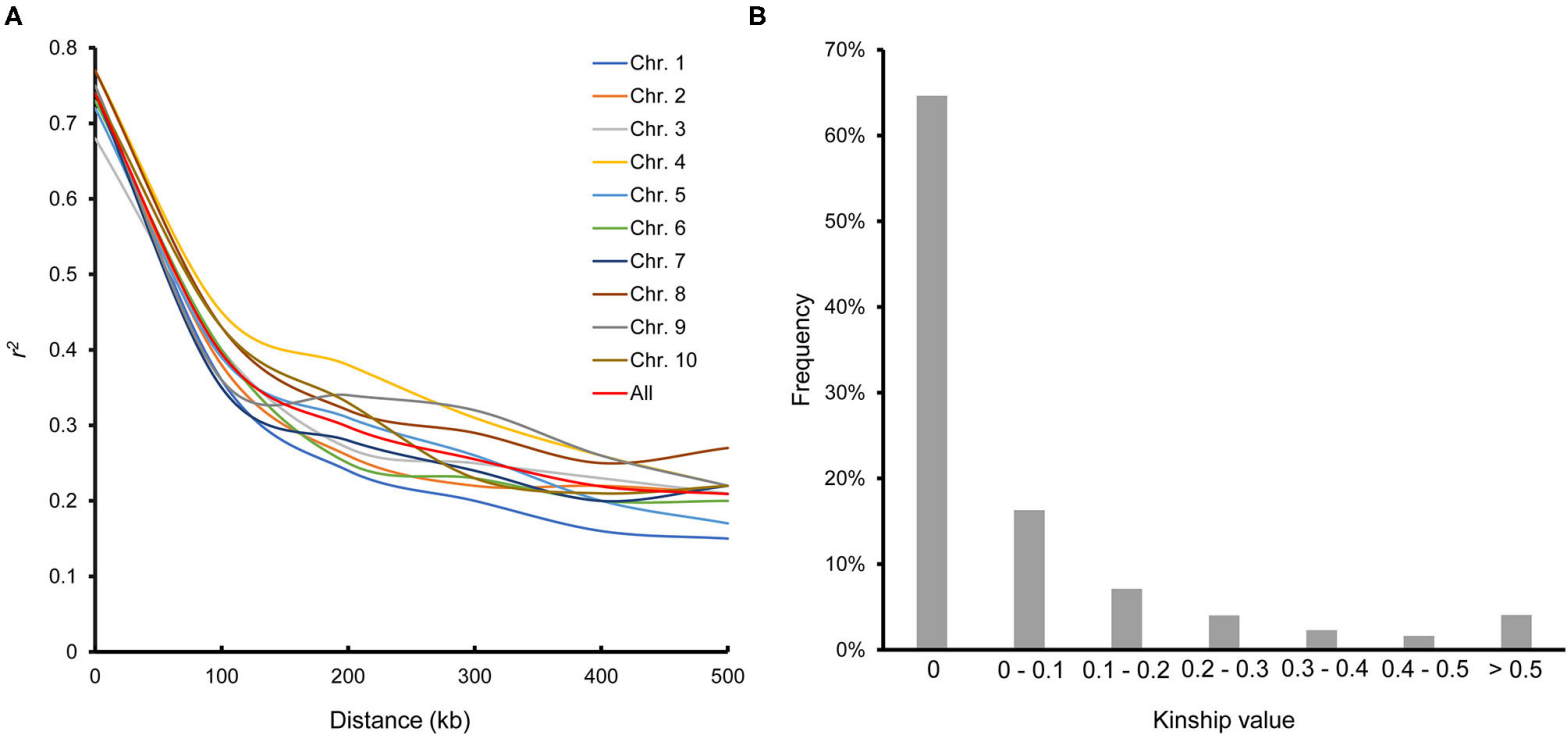
Linkage disequilibrium across the 10 chromosomes **(A)** and pairwise relative kinship for 251 maize inbred lines **(B)**.

LD decayed differently in the 10 chromosomes; chromosome 7 had the most rapid decay rate, and chromosome 4 had the slowest. The average LD decay distance across all chromosomes was ~100 kb, where the LD parameter (*r*^2^) dropped to half of its maximum value (Figure 2A). The average pairwise relative kinship value was 0.076. Pairwise relative kinship values of 0 accounted for 64.64% of all kinship values, values from 0 to 0.2 accounted for 88.03%, and values >0.5 accounted for only 4.07% (Figure 2B). This result revealed that the 251 inbred lines used in this study were distantly related.

### Phenotypic Variation in KMC

**Figure 3:**
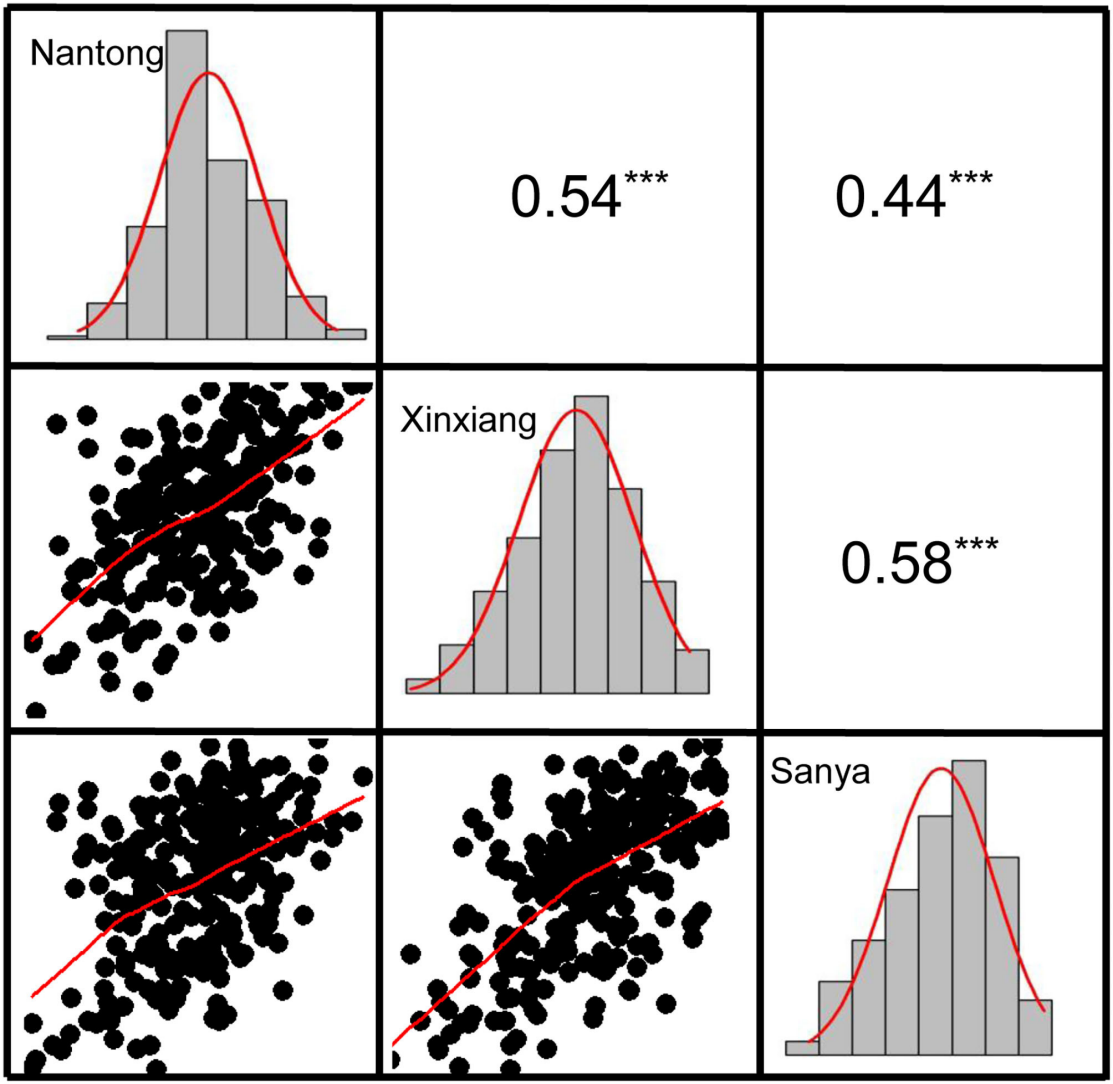
The correlation and frequency distribution of KMC in three environments. The upper panel is correlation coefficients, and the lower panel is scatter plots. The histogram represents the frequency distribution of the trait. ***the significance level at *P* < 0.001.

Descriptive statistics for KMC are presented in Table 1. KMC showed abundant variation among the 251 lines in each environment and was positively correlated among the different environments (Figure 3). The KMC in each environment approximately fitted a normal distribution with small skewness and kurtosis and high W-value (*P* > 0.05) (Table 1; Figure 3), suggesting that KMC was controlled by multiple genetic loci in this association mapping panel. ANOVA showed that the environment exerted significant influence on KMC (Supplementary Table 2), and the heritability was high (67.36–75.86%) (Table 1).

**Table 1:** Phenotypic performance, variance component and heritability of KMC.

**Env. ^a^**	**Mean ± SD^b^ (%)**	**Range (%)**	**Skewness**	**Kurtosis**	**W-value**	**$\sigma_{\text{g}}^{2c}$**	**$\sigma_{\text{ge}}^{2d}$**	***H^**2**^*(%)^e^**
Nantong	34.74 ± 6.23	18.63–51.13	−0.15	−0.17	0.99	25.60^**^		67.36
Xinxiang	35.30 ± 8.60	10.63–52.13	−0.29	−0.29	0.99	52.11^**^		70.42
Sanya	38.33 ± 8.03	18.42–53.83	−0.56	−0.42	0.97	44.63^**^		69.73
BLUP	36.12 ± 4.79	21.14–47.31	0.19	−0.42	0.99	30.20^**^	9.63^**^	75.86

a *Environment*.

b *Standard deviation*.

c *Variance of genotype*.

d *Variance of genotype × environment*.

e *Heritability*.

** *Significant at P < 0.01*.

**Figure 4:**
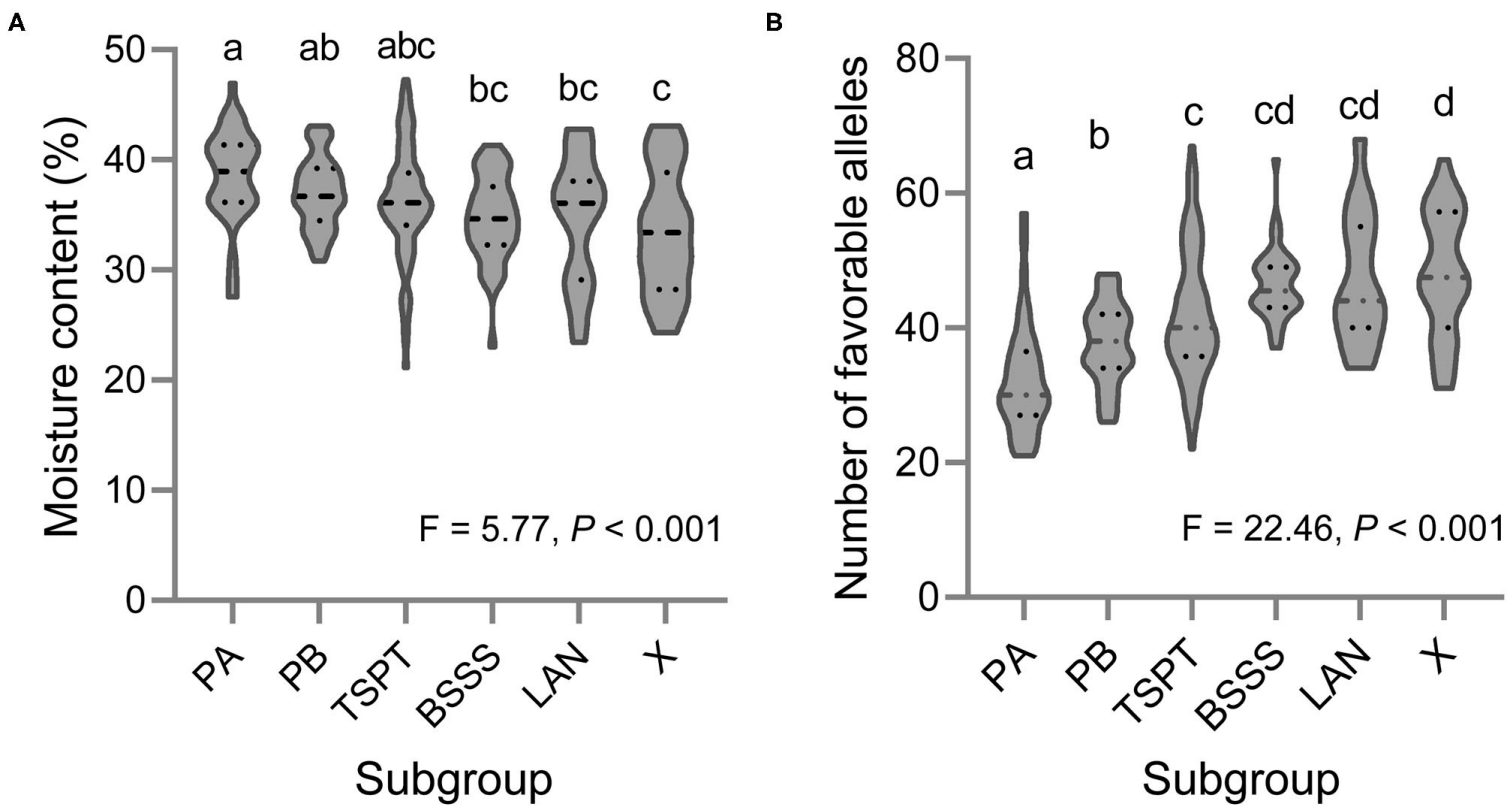
Violin plot of **(A)** the KMC and **(B)** the number of favorable alleles for KMC in six subpopulations of this association mapping panel. Different letters indicate significant difference at *P* < 0.001 estimated by Student's *t*-test.

Based on the BLUP values across the three environments, 11 of the 251 lines showed low KMC (below 27% moisture) (ID: 110, 111, 114, 131, 188, 209, 235, 242, 246, 247, and 249), and these are marked in bold in Supplementary Table 1. Fifty-five lines showed high KMC (above 40%), and the remaining lines showed moderate KMC (Supplementary Table 1). There was a significant difference in KMC among the six subpopulations; the KMC of subgroup X was the lowest, and that of subgroup PA was the highest (Figure 4A).

### Multi-Locus Genome-Wide Association Study of KMC

A total of 98 QTNs were detected in NT, XX, SY, and BLUP across all environments by the six ML-GWAS models, and each explained 0.67–26.96% of the phenotypic variation in KMC (Supplementary Table 3). Thirty-eight, 35, 23, 27, 34, and 39 QTNs were detected by mrMLM (Supplementary Figure 1), FASTmrMLM (Supplementary Figure 2), FASTmrEMMA (Supplementary Figure 3), PLARmEB, PKWmEB (Supplementary Figure 4), and ISIS EM-BLASSO, respectively. However, only 7 QTNs were detected in NT, XX, SY, and BLUP across all environments by the SL-GWAS model (MLM) (Supplementary Figure 5; Supplementary Table 3). Expect for *qKMC7.4*, the remaining 6 QTNs were overlapped with those from ML-GWAS models.

Among 98 QTNs, 44, 27, 16, 7, and 4 QTNs were co-detected by at least two, three, four, five, or six ML-GWAS models, respectively. 25, 25, 32, and 42 were detected in NT, XX, SY, and BLUP, respectively. However, no QTN was detected in three environments and BLUP, and only three QTNs (*qKMC2.15, qKMC6.1*, and *qKMC8.2*) were detected in two environments and BLUP.

Eleven QTNs were considered to be stable; these were distributed on chromosomes 1, 2, 3, 5, 6, 7, 8, and 10 (Table 2). Among the 11 stable QTNs, 11 were msQTNs, 1 was esQTNs, and 1 (*qKMC2.15*) was common between msQTNs and esQTNs. Seven of them overlapped previously reported genomic regions, and the remaining four were putatively novel loci. Only one QTN, *qKMC5.6*, accounted for more than 10% of the phenotypic variation (12.41–23.27%), and it may be a major genetic locus for KMC.

**Table 2:** Stable QTNs for KMC co-detected by at least four models under a uniform environment or in at least two environments and BLUP using the same ML-GWAS model.

**QTN**	**Marker**	**Chr. ^a^**	**Position (bp)**	**LOD**	***R*^**2**^ (%)^b^**	**Model^c^**	**Environment^d^**	**References**
*qKMC1.4*	AX-86284737	1	38082648	5.06–9.07	3.14–7.37	1, 2, 3, 4, 5, 6	XX, BLUP	Sala et al., 2012; Xiang et al., 2012
*qKMC1.5*	AX-86259253	1	39246603	3.50–9.41	3.02–7.66	1, 2, 3, 4, 5, 6	NT, BLUP	Sala et al., 2012; Xiang et al., 2012
*qKMC1.18*	AX-86266353	1	297863807	4.69–6.06	3.13–7.16	1, 2, 4, 5	XX, BLUP	
*qKMC2.15*	AX-116874459	2	178270600	5.15–7.42	2.19–6.23	1, 3, 4, 5, 6	NT, XX, BLUP	Xiang et al., 2012
*qKMC3.3*	AX-86264182	3	5148837	4.69–7.95	3.46–9.74	1, 2, 4, 5, 6	XX, BLUP	
*qKMC3.15*	AX-116872692	3	229667802	3.97–5.94	2.33–6.89	1, 2, 3, 4, 5, 6	XX, BLUP	Yin et al., 2020b
*qKMC5.6*	AX-86314969	5	61236323	4.21–7.84	12.41–23.27	1, 2, 4, 5, 6	NT	Xiang et al., 2012
*qKMC5.15*	AX-86282179	5	217125252	3.23–10.12	1.12–6.48	1, 2, 4, 6	NT, BLUP	Li et al., 2021
*qKMC6.7*	AX-86294630	6	163230474	3.41–7.67	3.29–7.93	1, 3, 4, 6	SY	
*qKMC8.3*	AX-86297230	8	174417551	3.09–5.97	2.45–3.89	2, 3, 4, 5	SY, BLUP	
*qKMC10.1*	AX-86257470	10	10313586	3.19–9.60	1.86–9.81	1, 2, 3, 4, 5, 6	XX, BLUP	Xiang et al., 2012

a *Chromosome*.

b *Phenotypic variation explained*.

c *1: mrMLM, 2: FASTmrMLM, 3: FASTmrEMMA, 4: PLARmEB, 5: PKWmEB, 6: ISIS EM-BLASSO*.

d *NT, Nantong; XX, Xinxiang; SY, Sanya; BLUP, best linear unbiased prediction*.

### Candidate Genes for KMC

According to the LD in this association panel (Figure 2A), 63 candidate genes were identified in 100 kb upstream and downstream of the 11 stable QTNs, and their expression in the kernel varied widely among 11 time points after pollination (Supplementary Table 4). Ten of these genes showed high expression (FPKM ≥ 20), which were marked in bold in Supplementary Table 4, suggesting that they may take part in the loss of kernel moisture. Nine of the genes encode proteins with assigned functions in multiple biological pathways, and the remaining gene encodes a protein of unknown function.

### Favorable Allele Mining

Based on the BLUP values across the three environments, the favorable alleles of 98 QTNs were mined (Supplementary Table 5). The inbred line ID. One hundred and ten harbors the most favorable alleles (69), and the inbred lines ID. Twenty one harbors the smallest favorable alleles (21). At group level, we found a significant negative correlation between the KMC and the number of favorable alleles in each inbred line (*R*^2^ = 0.68, slope = −0.41, intercept = 53.17, *P* < 0.001) using the linear regression analysis, indicating that pyramiding of these favorable alleles could reduce KMC effectively. There was a significant difference in number of favorable alleles among the six subpopulations; the number of favorable alleles of subgroup X was the most (48), and that of subgroup PA was the smallest (32) (Figure 4B).

### Genomic Selection of KMC

The average prediction accuracy was 0.12, 0.11, 0.13, and 0.17 in NT, XX, SY, and BLUP, respectively, when all 32,853 markers across the entire genome were included in model 1 (Table 3). To obtain optimal prediction accuracy, 12 different marker sets were included as fix effects in model 2. A significant increase in prediction accuracy was obtained when ML-GWAS-derived markers were used: the prediction accuracies based on markers detected by individual ML-GWAS model were almost the same and were approximately 0.34 in NT, 0.53 in XX, 0.29 in SY, and 0.59 in BLUP (Table 3). When all significant markers detected by all six ML-GWAS models were incorporated into the GS model, the prediction accuracy was highest: 0.54 in NT, 0.64 in XX, 0.58 in SY, and 0.76 in BLUP (Table 3).

**Table 3:** The prediction accuracy of the KMC when using markers in different ML-GWAS models in three environments and BLUP.

**Marker set^a^**	**Nantong**				**Xinxiang**				**Sanya**				**Best linear unbiased prediction**			
	***R***^**2**^**^b^**	**Slope**	**Intercept**	**RMSE^c^**	***R*^**2**^**	**Slope**	**Intercept**	**RMSE**	***R*^**2**^**	**Slope**	**Intercept**	**RMSE**	***R*^**2**^**	**Slope**	**Intercept**	**RMSE**
Genome	0.12	0.83	5.99	5.84	0.13	0.90	3.45	7.97	0.11	0.81	6.07	7.84	0.17	0.85	5.20	4.35
mrMLM	0.34	0.99	0.15	5.02	0.53	0.99	0.32	5.86	0.29	0.97	0.98	6.75	0.59	1.00	0.01	3.05
FASTmrMLM	0.34	0.99	0.37	5.02	0.53	0.99	0.28	5.86	0.29	0.98	0.77	6.75	0.59	1.00	0.12	3.06
FASTmrEMMA	0.34	0.99	0.19	5.01	0.53	1.00	0.19	5.85	0.29	0.98	0.76	6.73	0.59	1.00	0.05	3.06
PLARmEB	0.34	0.99	0.45	5.02	0.53	0.99	0.33	5.86	0.29	0.97	0.96	6.75	0.59	1.00	0.12	3.05
PKWmEB	0.34	0.99	0.23	5.02	0.53	0.99	0.32	5.85	0.29	0.97	1.14	6.76	0.59	1.00	0.08	3.05
ISIS EM-BLASSO	0.34	0.99	0.31	5.01	0.53	0.99	0.26	5.87	0.29	0.97	1.25	6.75	0.59	1.00	0.13	3.06
All models	0.54	1.01	−0.20	4.21	0.64	1.00	−0.06	5.12	0.58	1.02	−0.60	5.16	0.76	1.01	−0.29	2.32
C2	0.46	1.01	−0.31	4.55	0.60	1.00	−0.03	5.42	0.42	0.99	0.43	6.06	0.69	1.00	−0.05	2.64
C3	0.47	1.00	−0.05	4.48	0.54	0.99	0.33	5.81	0.33	0.98	0.33	6.52	0.65	0.99	0.05	2.81
C4	0.42	1.00	0.03	4.72	0.48	1.00	0.09	6.19	0.26	0.98	0.82	6.87	0.56	1.00	0.10	3.17
C5	0.19	0.98	0.86	5.59	0.20	0.99	0.51	7.42	0.14	0.95	1.21	7.81	0.22	0.96	1.10	4.21
C6	0.15	0.92	2.80	6.03	0.16	0.97	0.86	8.06	0.13	0.88	0.86	7.89	0.19	0.96	1.36	4.53

a *Genome indicated that 32,853 markers across the entire genome were included in model 1; mrMLM indicated that 38 markers identified by mrMLM model were included as fixed effects in model 2; FASTmrMLM indicated that 35 markers identified by FASTmrMLM model were included as fixed effects in model 2; FASTmrEMMA indicated that 23 markers identified by FASTmrEMMA model were included as fixed effects in model 2; PLARmEB indicated that 27 markers identified by PLARmEB model were included as fixed effects in model 2; PKWmEB indicated that 34 markers identified by PKWmEB model were included as fixed effects in model 2; ISIS EM-BLASSO indicated that 39 markers identified by ISIS EM-BLASSO model were included as fixed effects in model 2; All models indicated that 98 markers identified by all six ML-GWAS models were included as fixed effects in model 2; C2 indicated that 44 markers identified by at least two ML-GWAS models were included as fixed effects in model 2; C3 indicated that 27 markers identified by at least three ML-GWAS models were included as fixed effects in model 2; C4 indicated that 16 markers identified by at least four ML-GWAS models were included as fixed effects in model 2; C5 indicated that 7 markers identified by at least five ML-GWAS models were included as fixed effects in model 2; C6 indicated that 4 markers identified by at least six ML-GWAS models were included as fixed effects in model 2*.

b *Coefficient of determination*.

c *Square root of the mean square error*.

To explore whether using QTNs co-detected in multiple ML-GWAS models could improve prediction accuracy, we conducted GS using QTNs identified in at least two, three, four, five, or six models. Use of the QTNs identified in at least two, three, or four models maintain a relatively high prediction level, but QTNs identified in five or six models provided slight advantage in predicting KMC (Table 3). Seven QTNs were randomly selected from the 44 QTNs identified in at least two models (repeated 5 times) to conducted GS, and the mean of prediction accuracy was relatively low: 0.19 in NT, 0.15 in XX, 0.11 in SY, and 0.19 in BLUP, consistent with the results obtained by GS using QTNs identified in at least five models. This may be due to the smaller QTN numbers, which only explained a small fraction of phenotypic variance.

## Discussion

In this study, the 251 maize inbred lines were sown at three dates according to their growth periods, enabling us to measure KMC of each line over similar periods in each environment. The hand-held moisture meter was used to measure KMC, this is a reliable method and has been reported to be useful for evaluating genetic materials for QTL mapping (Sala et al., 2006; Kebede et al., 2016) and GWAS (Zhou et al., 2018; Li et al., 2021). However, we observed a significant genotype by environment interaction and relatively low correlation coefficients (0.44–0.58) among environments. This is typical because temperature, air humidity, and rainfall are uneven across environments, suggesting that genotype-by-environment interactions should be considered during maize breeding. Despite this issue, our aim was to obtain stable genetic loci that make a stable contribution to KMC. As described by Zhang et al. (2019), QTNs identified by multiple models are usually reliable when several ML-GWAS methods are applied to the same dataset. To reduce false positive signals and detect a set number of true positive loci, we considered two types of QTNs to be stable, one is msQTN, which is identified by at least four ML-GWAS models under an uniformed environment, and the other is esQTN, which is identified by in at least two environments and BLUP using the same ML-GWAS model.

Ninety-eight QTNs were identified by six ML-GWAS models in three environments and in BLUP across the three environments; eleven were considered to be stable QTNs. Only one QTN, *qKMC5.6*, explained more than 10% of the phenotypic variation, consistent with previous studies in which KMC was mainly controlled by numerous minor-effect genetic loci (Kebede et al., 2016; Song et al., 2017). Of these stable QTNs, 7 were located in genomic regions reported by previous studies, confirming the accuracy of QTN detection by ML-GWAS. Five QTNs (*qKMC1.4, qKMC1.5, qKMC2.15, qKMC5.6*, and *qKMC10.1*) were located in the meta-QTL regions estimated by Xiang et al. (2012) and Sala et al. (2012). In addition, *qKMC3.15* was located in the QTL regions reported by Yin et al. (2020b), and *qKMC5.15* overlapped with a QTL region identified by Li et al. (2021). More importantly, four novel genetic loci for KMC were identified in this study.

Candidate gene analysis of the stable QTNs is necessary for further gene cloning and functional verification. To date, only two genes underlying major QTLs for KMC have been identified (Li et al., 2021). In this study, 63 candidate genes were identified surrounding the 11 stable QTNs. Among these genes, 10 were highly expressed in the kernel at different time points after pollination, suggesting that they may potentially affect kernel water loss. We cannot accurately determine which are causal genes associated with KMC based on the present data. However, four of the candidate genes (*Zm00001d028560, Zm00001d005546, Zm00001d014742*, and *Zm00001d012439*) caught our attention.

*Zm00001d028560*, a candidate gene for *qKMC1.5*, encodes a leucine-rich repeat protein kinase that has been suggested to have an important role in signaling during pathogen recognition (Romeis, 2001; Afzal et al., 2007). Its homologous Arabidopsis gene, *FEI 1*, participates in cell wall elongation (Xu et al., 2008; Basu et al., 2016). Interestingly, this gene was located in the QTL regions for maize ear rot resistance identified by Butrón et al. (2019), Martin et al. (2012), and Robertson-Hoyt et al. (2007a). Previous studies have reported that KMC is negatively correlated with resistance to maize ear rot (Robertson-Hoyt et al., 2007b; Kebede et al., 2016). Moreover, Xiang et al. (2012) reported 14 pleiotropic meta-QTLs associated with both ear rot resistance and KMC in maize. These results suggest that *Zm00001d028560* may be simultaneously related to both KMC and ear rot resistance in maize.

*Zm00001d005546*, a candidate gene for *qKMC2.15*, encodes ADP-glucose pyrophosphorylase (AGPase), which provides the nucleotide sugar ADP-glucose and thus constitutes the first step in starch biosynthesis (Slattery et al., 2000; Comparot-Moss and Denyer, 2009). During the maize kernel filling period, AGPase activity and starch synthesis were significantly improved by increasing AGPase expression (Ozbun et al., 1973; Li et al., 2010). In addition, *Zm00001d005546* is located in a QTL region related to the maize kernel filling process identified by Yin et al. (2020a). Kernel filling had a notable influence on kernel drying rate before and after physiological maturity in maize (Jia et al., 2020). Therefore, we hypothesize that high expression of *Zm00001d005546* may have promoted starch synthesis in the kernel, increasing kernel filling rate, and thereby accelerating kernel dehydration rate before physiological maturity. This may have prolonged the field dehydration time of the kernel and ultimately resulted in low KMC. This explanation also provides a molecular hypothesis for the maize breeding phenomenon in which a hybrid or inbred line with high kernel filling rate generally has low KMC (Johnson and Tanner, 1972; Kang and Zuber, 1989). More experiments are needed to elucidate the function and mechanism of *Zm00001d005546*.

*Zm00001d014742* encodes F-box domain protein and is a candidate gene for the major QTN, *qKMC5.6*. Its homologous Arabidopsis gene, *AtSKIP31*, involves in primary root growth under nitrogen deficiency and regulates the nitrogen utilization efficiencies (Hong et al., 2017). Nitrogen utilization efficiencies are related to grain yield and maturation (Wang W. et al., 2018), which have positive correlation with the KMC (Zhou et al., 2018; Li et al., 2021). *Zm00001d012439*, a candidate gene for the novel QTN, *qKMC8.3*, encodes histone H4, which may affect gene transcription activity through histone modification (Heintz, 1991).

In this study, 251 maize inbred lines were divided into six subgroups, that was, PA, PB, TSPT, BASSS, LAN and X. Among these six subgroups, Subgroup PA had highest KMC and the least number of favorable alleles for the KMC, however, Subgroup PA contained multiple elite inbred lines, such as Ye478 and Zheng58, and have played an important role in maize breeding in China over the last 40 years (Li and Wang, 2010). Subgroup X had lowest KMC and the greatest number of favorable alleles for the KMC, and has being gradually applied in maize breeding in China (Zhao et al., 2018). This phenomenon is mainly due to the changes of maize breeding goals at different periods in China. Before 2010, maize harvesting mainly relied on manual. Farmers preferred to plant the varieties with large ear under low density condition to ensure yield. Chinese maize breeders increased yields by extending the growth period. Subgroup PA had the characteristics of large ears and long growth period, which was in line with the breeding goals at that time (Li and Wang, 2010). Maize inbred line or hybrid with large ear and long growth period tended to high KMC (Zhou et al., 2018; Li et al., 2021). In recent years, with the development of agricultural modernization, mechanical harvesting of grain is the developing direction of maize production (Li et al., 2017). Chinese maize breeders have increasingly concentrated on the KMC. Thus, a new heterosis group, Subgroup X, was breed. Subgroup X had short growth period and low KMC, which was suitable for mechanical harvesting of maize grain (Zhao et al., 2018).

With advances in sequencing technology and reduction of testing costs, GS has been widely implemented in plant breeding. Fitting GS models need to face the fact that the number of markers (*p*) far exceeds the number of individuals (*n*) (de los Campos et al., 2013). Consequently, when a GS model that considers the additive effect of each marker is fitted to such large *p* and small *n* data, there will be an infinite number of maximum likelihood estimates of these effects (Gianola, 2013). rrBLUP incorporates all marker information to predict an individual genomic estimated value while simultaneously implementing a penalization function to restrict the values that each marker predicted additive contributions can equal, which is an effective model to overcome this issue (Meuwissen et al., 2001). In this study, the *p* is 32,853, while the *n* is only 251. Thus, rrBLUP was selected to conduct GS. When using 32,853 markers across the entire genome, we obtained a lower prediction accuracy (0.11–0.17). However, higher prediction levels were easily attained when using the ML-GWAS-derived markers included as fix effects. The prediction accuracy was still high (0.26–0.56) when only 16 stable markers identified by at least four models were included. Similar findings were reported for maize kernel row number (An et al., 2020), resistance to maize southern leaf blight and gray leaf spot (Bian and Holland, 2017), and maize low-phosphorus tolerance (Xu et al., 2018). Therefore, using a small set of markers identified by multiple ML-GWAS methods as fixed effects in an rrBLUP model is a powerful tool for KMC prediction in maize molecular breeding and can effectively save time and costs.

## Conclusions

Ninety-eight QTNs for KMC were identified using six ML-GWAS models in three environments and BLUP across three environments. Eleven QTNs were considered to be stable. Seven stable QTNs corresponded to previously reported QTL regions, whereas the remaining four were putatively novel loci. Sixty-three candidate genes were identified within LD blocks of the 11 stable QTNs. Among these candidates, 10 may potentially affect the loss of water from the maize kernel. High prediction levels were easily reached when the KMC-associated markers were included as fixed effects in GS. The best strategy was to integrate all KMC-associated markers identified by all six ML-GWAS models. These results facilitate our understanding of the genetic basis of KMC and provide useful information for the reduction of KMC in maize breeding.

## Data Availability Statement

The original contributions presented in the study are included in the article/Supplementary Material, further inquiries can be directed to the corresponding author/s.

## Author Contributions

GZ and DH designed the experiment and wrote the manuscript. GZ, YM, LX, GC, ZZ, XS, HZ, HL, MS, and DH performed the experiments and collected the phenotypic data. GZ and QZ analyzed the genotypic and phenotypic data. All authors read and approved the final manuscript.

## Conflict of Interest

The authors declare that the research was conducted in the absence of any commercial or financial relationships that could be construed as a potential conflict of interest.

## Supplementary Material

Supplementary Figure 1 Manhattan (left) and quartile-quartile (right) plots of ML-GWAS results using mrMLM model. The four circles of Manhattan plot from inside to outside show the result in Nantong (NT), Xinxiang (XX), Sanya (SY), and best linear unbiased prediction (BLUP), respectively.

Supplementary Figure 2 Manhattan (left) and quartile-quartile (right) plots of ML-GWAS results using FASTmrMLM model. The four circles of Manhattan plot from inside to outside show the result in Nantong (NT), Xinxiang (XX), Sanya (SY), and best linear unbiased prediction (BLUP), respectively.

Supplementary Figure 3 Manhattan (left) and quartile-quartile (right) plots of ML-GWAS results using FASTmrEMMA model. The four circles of Manhattan plot from inside to outside show the result in Nantong (NT), Xinxiang (XX), Sanya (SY), and best linear unbiased prediction (BLUP), respectively.

Supplementary Figure 4 Manhattan (left) and quartile-quartile (right) plots of ML-GWAS results using PKWmEB model. The four circles of Manhattan plot from inside to outside show the result in Nantong (NT), Xinxiang (XX), Sanya (SY), and best linear unbiased prediction (BLUP), respectively.

Supplementary Figure 5 Manhattan (left) and quartile-quartile (right) plots of SL-GWAS results using MLM model. The four circles of Manhattan plot from inside to outside show the result in Nantong (NT), Xinxiang (XX), Sanya (SY), and best linear unbiased prediction (BLUP), respectively.

Supplementary Table 1 The origin or pedigree, subpopulation, sowing data, and phenotype of 251 maize inbred lines used in this study.

Supplementary Table 2 Analysis of variance for KMC across three environments.

Supplementary Table 3 The significant QTNs for KMC identified by MLM and six ML-GWAS models in this study.

Supplementary Table 4 Candidate genes around the 11 stable QTNs for KMC and their FPKM expression data in the whole kernle of B73 at different days after pollination (DAP).

Supplementary Table 5 The distribution of favorable alleles for 98 QTNs in 251 maize inbred lines.

## References

[B1] Afzal A. J. Wood A. J. Lightfoot D. A. (2007). Plant receptor-like serine threonine kinases: roles in signaling and plant defense. Mol. Plant Microbe Interact. 21, 507–517. 10.1094/MPMI-21-5-0507 18393610

[B2] An Y. X. Chen L. Li Y. X. Li C. H. Shi Y. S. Zhang D. F.. (2020). Genome-wide association studies and whole-genome prediction reveals the genetic architecture of KRN in maize. BMC Plant Biol. 20:490. 10.1186/s12870-020-02676-x 33109077 PMC7590725

[B3] Austin D. F. Lee M. Veldboom L. R. Hallauer A. R. (2000). Genetic mapping in maize with hybrid progeny across testers and generations: grain yield and grain moisture. Crop Sci. 40, 30–39. 10.2135/cropsci2000.40130x26731606

[B4] Basu S. Tian L. Debrosse T. Poirier E. Emch K. Herock H.. (2016). Glycosylation of a fasciclin-like arabinogalactan-protein (SOS5) mediates root growth and seed mucilage adherence via a cell wall receptor-like kinase (FEI1/FEI2) pathway in Arabidopsis. PLoS ONE 11:e0145092. 10.1371/journal.pone.0145092 26731606 PMC4701510

[B5] Beavis W. D. Smith O. S. Grant D. Fincher R. (1994). Identification of quantitative trait loci using a small sample of topcrossed and F_4_ progeny from maize. Crop Sci. 34, 882–896. 10.2135/cropsci1994.0011183X003400040010x

[B6] Bian Y. Holland J. B. (2017). Enhancing genomic prediction with genome-wide association studies in multiparental maize populations. Heredity 118, 585–593. 10.1038/hdy.2017.4 28198815 PMC5436027

[B7] Blanc G. Charcosset A. Mangin B. Gallais A. Moreau L. (2006). Connected populations for detecting quantitative trait loci and testing for epistasis: an application in maize. Theor. Appl. Genet. 113, 206–224. 10.1007/s00122-006-0287-1 16791688

[B8] Bradbury P. J. Zhang Z. Kroon D. E. Casstevens T. M. Ramdoss Y. Buckler E. S. (2007). TASSEL: software for association mapping of complex traits in diverse samples. Bioinformatics 23, 2633–2635. 10.1093/bioinformatics/btm308 17586829

[B9] Brooking I. P. (1990). Maize ear moisture during grain-filling, and its relation to physiological maturity and grain-drying. Field Crops Res. 23, 55–68. 10.1016/0378-4290(90)90097-U

[B10] Butrón A. Santiago R. Cao A. Samayoa L. F. Malvar R. A. (2019). QTLs for resistance to Fusarium ear rot in a multi-parent advanced generation inter-cross (MAGIC) of maize population. Plant Dis. 103, 897–904. 10.1094/PDIS-09-18-1669-RE 30856072

[B11] Capelle V. Remoué C. Moreau L. Reyss A. Mahé A. Massonneau A.. (2010). QTLs and candidate genes for desiccation and abscisic acid content in maize kernels. BMC Plant Biol. 10:2. 10.1186/1471-2229-10-2 20047666 PMC2826337

[B12] Comparot-Moss S. Denyer K. (2009). The evolution of the starch biosynthetic pathways in cereals and other grasses. J. Exp. Bot. 60, 2481–2492. 10.1093/jxb/erp141 19505928

[B13] de los Campos G. Hickey J. M. Pong-Wong R. Daetwyler H. D. Calus M. P. L. (2013). Whole-genome regression and prediction methods applied to plant and animal breeding. Genetics 193, 327–345. 10.1534/genetics.112.143313 22745228 PMC3567727

[B14] Endelman J. B. (2011). Ridge regression and other kernels for genomic selection with R package rrBLUP. Plant Genome 4, 250–255. 10.3835/plantgenome2011.08.0024

[B15] Evanno G. Regnaut S. Goudet J. (2005). Detecting the number of clusters of individuals using the software STRUCTURE: a simulation study. Mol. Ecol. 14, 2611–2620. 10.1111/j.1365-294X.2005.02553.x 15969739

[B16] Frascaroli E. Canè M. Landi P. Pea G. Gianfranceschi L. Villa M.. (2007). Classical genetic and quantitative trait loci analyses of heterosis in a maize hybrid between two elite inbred lines. Genetics 176:625. 10.1534/genetics.106.064493 17339211 PMC1893040

[B17] Gianola D. (2013). Priors in whole-genome regression: the Bayesian alphabet returns. Genetics 194, 573–596. 10.1534/genetics.113.151753 23636739 PMC3697965

[B18] Hardy O. J. Vekemans X. (2002). SPAGeDi: a versatile computer program to analyse spatial genetic structure at the individual or population levels. Mol. Ecol. Res. 2, 618–620. 10.1046/j.1471-8286.2002.00305.x

[B19] Heintz N. (1991). The regulation of histone gene expression during the cell cycle. Biochim. Biophys. Acta 1088, 327–339. 10.1016/0167-4781(91)90122-3 2015297

[B20] Ho J. Mccouch S. Smith M. (2002). Improvement of hybrid yield by advanced backcross qtl analysis in elite maize. Theor. Appl. Genet. 105:440. 10.1007/s00122-002-0945-x 12582549

[B21] Hong J. P. Adams E. Yanagawa Y. Matsui M. Shin R. (2017). AtSKIP18 and AtSKIP31, F-box subunits of the SCF E3 ubiquitin ligase complex, mediate the degradation of 14-3-3 proteins in *Arabidopsis*. Biochem. Bioph. Res. Co. 485, 174–180. 10.1016/j.bbrc.2017.02.046 28189687

[B22] Jia T. J. Wang L. F. Li J. J. Ma J. Cao Y. Y. Lübberstedt T.. (2020). Integrating a genome-wide association study with transcriptomic analysis to detect genes controlling grain drying rate in maize (*Zea may* L.). Theor. Appl. Genet. 133, 623–634. 10.1007/s00122-019-03492-0 31797010

[B23] Johnson D. R. Tanner J. W. (1972). Calculation of the rate and duration of grain filling in corn (*Zea mays* L.). Crop Sci. 12, 485–486. 10.2135/cropsci1972.0011183X001200040028x

[B24] Kang M. S. Zuber M. S. (1989). Combining ability for grain moisture, husk moisture, and maturity in maize with yellow and white endosperms. Crop Sci. 29, 689–692. 10.2135/cropsci1989.0011183X002900030030x

[B25] Kebede A. Z. Woldemariam T. Reid L. M. Harris L. J. (2016). Quantitative trait loci mapping for Gibberella ear rot resistance and associated agronomic traits using genotyping-by-sequencing in maize. Theor. Appl. Genet. 129, 17–29. 10.1007/s00122-015-2600-3 26643764

[B26] Knapp S. J. Stroup W. W. Ross W. M. (1985). Exact confidence intervals for heritability on a progeny mean basis. Crop Sci. 25, 192–194. 10.2135/cropsci1985.0011183X002500010046x

[B27] Kumar S. Stecher G. Li M. Knyaz C. Tamura K. (2018). MEGA X: molecular evolutionary genetics analysis across computing platforms. Mol. Biol. Evol. 35, 1547–1549. 10.1093/molbev/msy096 29722887 PMC5967553

[B28] Li L. L. Lei X. P. Xie R. Z. Wang K. R. Hong P. Zhang F. G.. (2017). Analysis of influential factors on mechanical grain harvest quality of summer maize. Sci. Agric. Sin. 50, 2044–2051. 10.3864/j.issn.0578-1752.2017.11.010

[B29] Li L. L. Xie R. Z. Fan P. P. Lei X. P. Wang K. R. Hou P.. (2016). Study on dehydration in kernel between Zhengdan958 and Xianyu335. J. Maize Sci. 24, 57–61. 10.13597/j.cnki.maize.science.20160212

[B30] Li N. Zhang S. J. Zhao Y. J. Li B. Zhang J. R. (2010). Over-expression of AGPase genes enhances seed weight and starch content in transgenic maize. Planta 233, 241–250. 10.1007/s00425-010-1296-5 20978801

[B31] Li W. Q. Yu Y. H. Wang L. X. Luo Y. Peng Y. Xu Y. C.. (2021). The genetic architecture of the dynamic changes in grain moisture in maize. Plant Biotechnol. J. 19, 1195–1205. 10.1111/pbi.13541 33386670 PMC8196655

[B32] Li Y. Wang T. Y. (2010). Germplasm base of maize breeding in China and formation of foundation parents. J. Maize Sci. 18, 1–8.

[B33] Liu F. H. Wang K. R. Jian L. Wang X. M. Sun Y. L. Chen Y. S.. (2013). Factors affecting corn mechanically harvesting grain quality. Crops 4, 116–119.

[B34] Liu J. J. Yu H. Liu Y. L. Deng S. N. Liu Q. C. Liu B. S.. (2020). Genetic dissection of grain water content and dehydration rate related to mechanical harvest in maize. BMC Plant Biol. 20:118. 10.1186/s12870-020-2302-0 32183696 PMC7076969

[B35] Martin M. Miedaner T. Schwegler D. D. Kessel B. Ouzunova M. Dhillon B. S.. (2012). Comparative quantitative trait loci mapping for Gibberella ear rot resistance and reduced deoxynivalenol contamination across connected maize populations. Crop Sci. 52, 32–43. 10.2135/cropsci2011.04.0214

[B36] Melchinger A. E. Utz H. F. Schön C. C. (1998). Quantitative trait locus (QTL) mapping using different testers and independent population samples in maize reveals low power of QTL detection and large bias in estimates of QTL effects. Genetics 149, 383–403. 10.1093/genetics/149.1.383 9584111 PMC1460144

[B37] Meuwissen T. H. Hayes B. J. Goddard M. E. (2001). Prediction of total genetic value using genome-wide dense marker maps. Genetics 157, 1819–1829. 10.1093/genetics/157.4.1819 11290733 PMC1461589

[B38] Mihaljevic R. Utz H. F. Melchinger A. E. (2004). Congruency of quantitative trait loci detected for agronomic traits in testcrosses of five populations of European maize. Crop Sci. 44, 114–124. 10.2135/cropsci2004.1140

[B39] Mihaljevic R. Utz H. F. Melchinger A. E. (2005). No evidence for epistasis in hybrid and per se performance of elite European flint maize inbreds from generation means and qtl analyses. Crop Sci. 45, 2605–2613. 10.2135/cropsci2004.0760

[B40] Nei M. F. (1972). Genetic distance between populations. Am. Nat. 106, 283–292. 10.1086/282771

[B41] Ozbun J. L. Hawker J. S. Greenberg E. Lammel C. Preiss J. (1973). Starch synthetase, phosphorylase, ADPglucose pyrophyosphortlase, and UDPglucose pyrophyosphortlase in developing maize kernels. Plant Physiol. 51, 1–5. 10.1104/pp.51.1.1 16658267 PMC367346

[B42] Pritchard J. K. Stephens M. Donnelly P. (2000). Infernece of population structure using multilocus gentoype data. Genetics 155, 945–959. 10.1093/genetics/155.2.945 10835412 PMC1461096

[B43] Purcell S. Neale B. Todd-Brown K. Thomas L. Ferreira M. A. Bender D.. (2007). PLINK: a tool set for whole-genome association and population-based linkage analysis. Am. J. Hum. Genet. 81, 559–575. 10.1086/519795 17701901 PMC1950838

[B44] Qin J. Shi A. N. Song Q. J. Li S. Wang F. M. Cao Y. D.. (2019). Genomic-wide association study and genomic selection of amino acid concentrations in soybean seeds. Front. Plant Sci. 10:1445. 10.3389/fpls.2019.01445 31803203 PMC6873630

[B45] Ravelombola W. S. Qin J. Shi A. N. Nice L. Bao Y. Lorenz A.. (2019). Genome-wide association study and genomic selection for soybean chlorophyll content associated with soybean cyst nematode tolerance. BMC Genomics 20:904. 10.1186/s12864-019-6275-z 31775625 PMC6882315

[B46] Reid L. M. Zhu X. Morrison M. J. Woldemariam T. Voloaca C. Wu J.. (2010). A non-destructive method for measuring maize kernel moisture in a breeding program. Maydica 55, 163–171. 10.3198/jpr2009.06.0350crmp

[B47] Ren W. L. Wen Y. J. Dunwell J. M. Zhang Y. M. (2018). pKWmEB: integration of Kruskal-Wallis test with empirical bayes under polygenic background control for multi-locus genome-wide association study. Heredity 120, 208–218. 10.1038/s41437-017-0007-4 29234158 PMC5836593

[B48] Roberston-Hoyt L. A. Kleinschmidt C. E. White D. G. Payne G. A. Maragos C. M. Holland J. B. (2007b). Relationships of resistance to Fusarium ear rot and Fumonisin contamination with agronomic performance of maize. Crop Sci. 47, 1770–1778. 10.2135/cropsci2006.10.0676

[B49] Robertson-Hoyt L. A. Betrán J. Payne G. A. White D. G. Isakeit T. Maragos C. M.. (2007a). Relationships among resistances to Fusarium and Aspergillus ear rots and contamination by fumonisin and aflatoxin in maize. Phytopathology 97, 311–317. 10.1094/PHYTO-97-3-0311 18943650

[B50] Romeis T. (2001). Protein kinases in the plant defence response. Curr. Opin. Plant Biol. 4, 407–414. 10.1016/S1369-5266(00)00193-X 11597498

[B51] Sala R. G. Andrade F. H. Camadro E. L. Cerono J. C. (2006). Quantitative trait loci for grain moisture at harvest and field grain drying rate in maize (*Zea mays* L.). Theor. Appl. Genet. 112, 462–471. 10.1007/s00122-005-0146-5 16311725

[B52] Sala R. G. Andrade F. H. Cerono J. C. (2012). Quantitative trait loci associated with grain moisture at harvest for line *per se* and testcross performance in maize: a meta-analysis. Euphytica 185, 429–440. 10.1007/s10681-011-0614-8

[B53] Sehgal D. Rosyara U. Mondal S. Singh R. Poland J. Dreisigacker S. (2020). Incorporating genome-wide association mapping results into genomic prediction models for grain yield and yield stability in CIMMYT spring bread wheat. Front Plant Sci. 11:197. 10.3389/fpls.2020.00197 32194596 PMC7064468

[B54] Slattery C. J. Kavakli I. H. Okita T. W. (2000). Engineering starch for increased quantity and quality. Trends Plant Sci. 5, 291–298. 10.1016/S1360-1385(00)01657-5 10871901

[B55] Song W. Shi Z. Xing J. F. Duan M. X. Su A. G. Li C. H.. (2017). Molecular mapping of quantitative trait loci for grain moisture at harvest in maize. Plant Breed. 136, 28–32. 10.1111/pbr.1243031725912

[B56] Spindel J. E. Begum H. Akdemir D. Collard B. Redona E. Jannink J. L.. (2016). Genome-wide prediction models that incorporate *de novo* GWAS are a powerful new tool for tropical rice improvement. Heredity 116, 395–408. 10.1038/hdy.2015.113 26860200 PMC4806696

[B57] Tamba C. L. Ni Y. L. Zhang Y. M. (2017). Iterative sure independence screening EM-Bayesian LASSO algorithm for multi-locus genome-wide association studies. PLoS Comput. Biol. 13:e1005357. 10.1371/journal.pcbi.1005357 28141824 PMC5308866

[B58] Tamba C. L. Zhang Y. M. (2018). A fast mrMLM algorithm for multi-locus genome-wide association studies. bioRxiv [Preprint]. 10.1101/341784

[B59] Wang S. B. Feng J. Y. Ren W. L. Huang B. Zhou L. Wen Y. J.. (2016). Improving power and accuracy of genome-wide association studies via a multi-locus mixed linear model methodology. Sci. Rep. 6:19444. 10.1038/srep19444 26787347 PMC4726296

[B60] Wang W. Hu B. Yuan D. Y. Liu Y. Q. Che R. H. Hu Y. C.. (2018). Expression of the nitrate transporter gene *OsNRT1.1A/OsNPF6.3* confers high yield and early maturation in rice. Plant Cell 30, 638–651. 10.1105/tpc.17.00809 29475937 PMC5894839

[B61] Wang X. Xu Y. Hu Z. L. Xu C. W. (2018). Genomic selection methods for crop improvement: current status and prospects. Crop J. 6, 330–340. 10.1016/j.cj.2018.03.001

[B62] Wen Y. J. Zhang H. Ni Y. L. Huang B. Zhang J. Feng J. Y.. (2018). Methodological implementation of mixed linear models in multi-locus genome-wide association studies. Brief. Bioinform. 19, 700–712. 10.1093/bib/bbw145 28158525 PMC6054291

[B63] Xiang K. Reid L. M. Zhang Z. M. Zhu X. Y. Pan G. T. (2012). Characterization of correlation between grain moisture and ear rot resistance in maize by QTL meta-analysis. Euphytica 183, 185–195. 10.1007/s10681-011-0440-z

[B64] Xu C. Zhang H. W. Sun J. H. Guo Z. F. Zou C. Li W. X.. (2018). Genome-wide association study dissects yield components associated with low-phosphorus stress tolerance in maize. Theor. Appl. Genet. 131, 1699–1714. 10.1007/s00122-018-3108-4 29754325

[B65] Xu S. L. Rahman A. Baskin T. I. Kieber J. J. (2008). Two leucine-rich repeat receptor kinases mediate signaling, linking cell wall biosynthesis and ACC synthase in *Arabidopsis*. Plant Cell 20, 3065–3079. 10.1105/tpc.108.063354 19017745 PMC2613664

[B66] Xu Y. B. Liu X. G. Fu J. J. Wang H. W. Wang J. K. Huang C. L.. (2020). Enhancing genetic grain through genomic selection: from livestock to plants. Plant Comm. 1:100005. 10.1016/j.xplc.2019.100005 PMC7747995 33404534

[B67] Yin S. Y. Li P. C. Xu Y. Liu J. Yang T. T. Wei J.. (2020a). Genetic and genomic analysis of the seed-filling process in maize based on a logistic model. Heredity 124, 122–134. 10.1038/s41437-019-0251-x 31358987 PMC6906428

[B68] Yin S. Y. Liu J. Yang T. T. Li P. C. Xu Y. Fang H. M.. (2020b). Genetic analysis of the seed dehydration process in maize based on a logistic model. Crop J. 8, 182–193. 10.1016/j.cj.2019.06.011 PMC6906428 31358987

[B69] Zhang J. Feng J. Y. Ni Y. L. Wen Y. J. Niu Y. Tamba C. L.. (2017). pLARmEB: integration of least angle regression with empirical bayes for multi-locus genome-wide association studies. Heredity 118, 517–524. 10.1038/hdy.2017.8 28295030 PMC5436030

[B70] Zhang J. Zhang F. Q. Tang B. J. Ding Y. Xia L. K. Qi J. S.. (2020). Molecular mapping of quantitative trait loci for grain moisture at harvest and field grain drying rat in maize (*Zea mays* L.). Physiol. Plant. 169, 64–72. 10.1111/ppl.13048 31725912

[B71] Zhang Y. L. Liu P. Zhang X. X. Zheng Q. Chen M. Ge F.. (2018). Multi-locus genome-wide association study reveals the genetic architecture of stalk lodging resistance-related traits in maize. Front. Plant Sci. 9:611. 10.3389/fpls.2018.00611 29868068 PMC5949362

[B72] Zhang Y. M. Jia Z. Dunwell J. M. (2019). Editorial: the application of new multi-locus GWAS methodologies in the genetic dissection of complex traits. Front. Plant Sci. 10:100. 10.3389/fpls.2019.00100 30804969 PMC6378272

[B73] Zhao J. R. Li C. H. Song W. Wang Y. D. Zhang R. Y. Wang J. D.. (2018). Genetic diversity and population structure of important Chinese maize breeding germplasm revealed by SNP-Chips. Sci. Agric. Sin. 51, 626–634. 10.3864/j.issn.0578-1752.2018.04.003

[B74] Zhou G. F. Hao D. R. Chen G. Q. Lu H. H. Shi M. L. Mao Y. X.. (2016). Genome-wide association study of the husk number and weight in maize (*Zea mays* L.). Euphytica 210, 195–205. 10.1007/s10681-016-1698-y

[B75] Zhou G. F. Hao D. R. Xue L. Chen G. Q. Lu H. H. Zhang Z. L.. (2018). Genome-wide association study of kernel moisture content at harvest stage in maize. Breed. Sci. 68, 622–628. 10.1270/jsbbs.18102 30697124 PMC6345239

[B76] Zhou G. F. Mao Y. X. Xue L. Chen G. Q. Lu H. H. Shi M. L.. (2020). Genetic dissection of husk number and length across multiple environments and fine-mapped of a major-effect QTL for husk number in maize. Crop J. 8, 1071–1080. 10.1016/j.cj.2020.03.009

