# Supplementary figures and images for: Multi-Locus Genome-Wide Association Study and Genomic Selection of Kernel Moisture Content at the Harvest Stage in Maize
Source: Front Plant Sci. 2021 Jul 9;12:697688. doi: 10.3389/fpls.2021.697688 (PMC8299107; doi:10.3389/fpls.2021.697688)

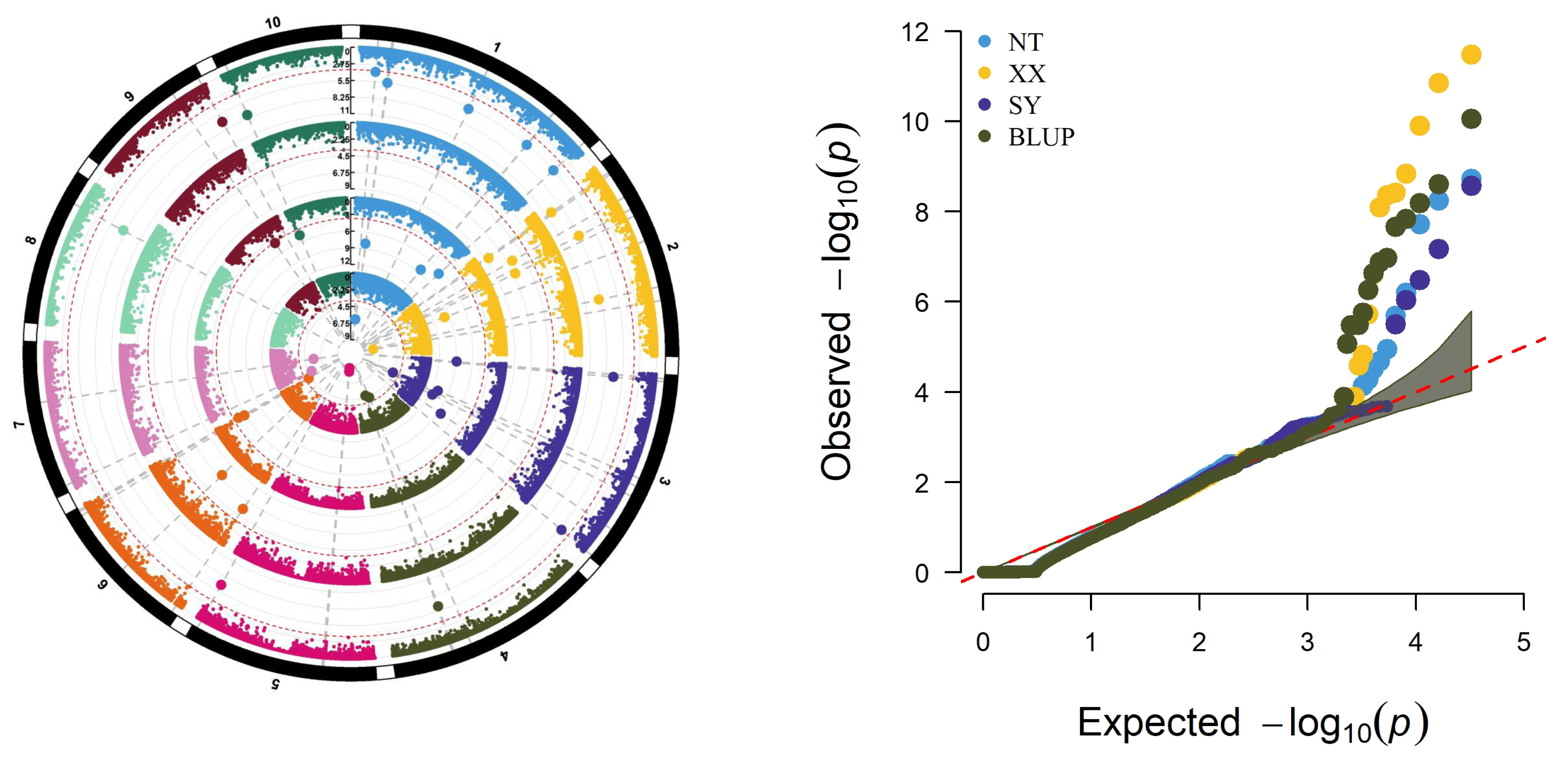

Supplement: Supplementary Figure 1 — Manhattan (left) and quartile-quartile (right) plots of ML-GWAS results using mrMLM model. The four circles of Manhattan plot from inside to outside show the result in Nantong (NT), Xinxiang (XX), Sanya (SY), and best linear unbiased prediction (BLUP), respectively. [file Image_1.TIF]

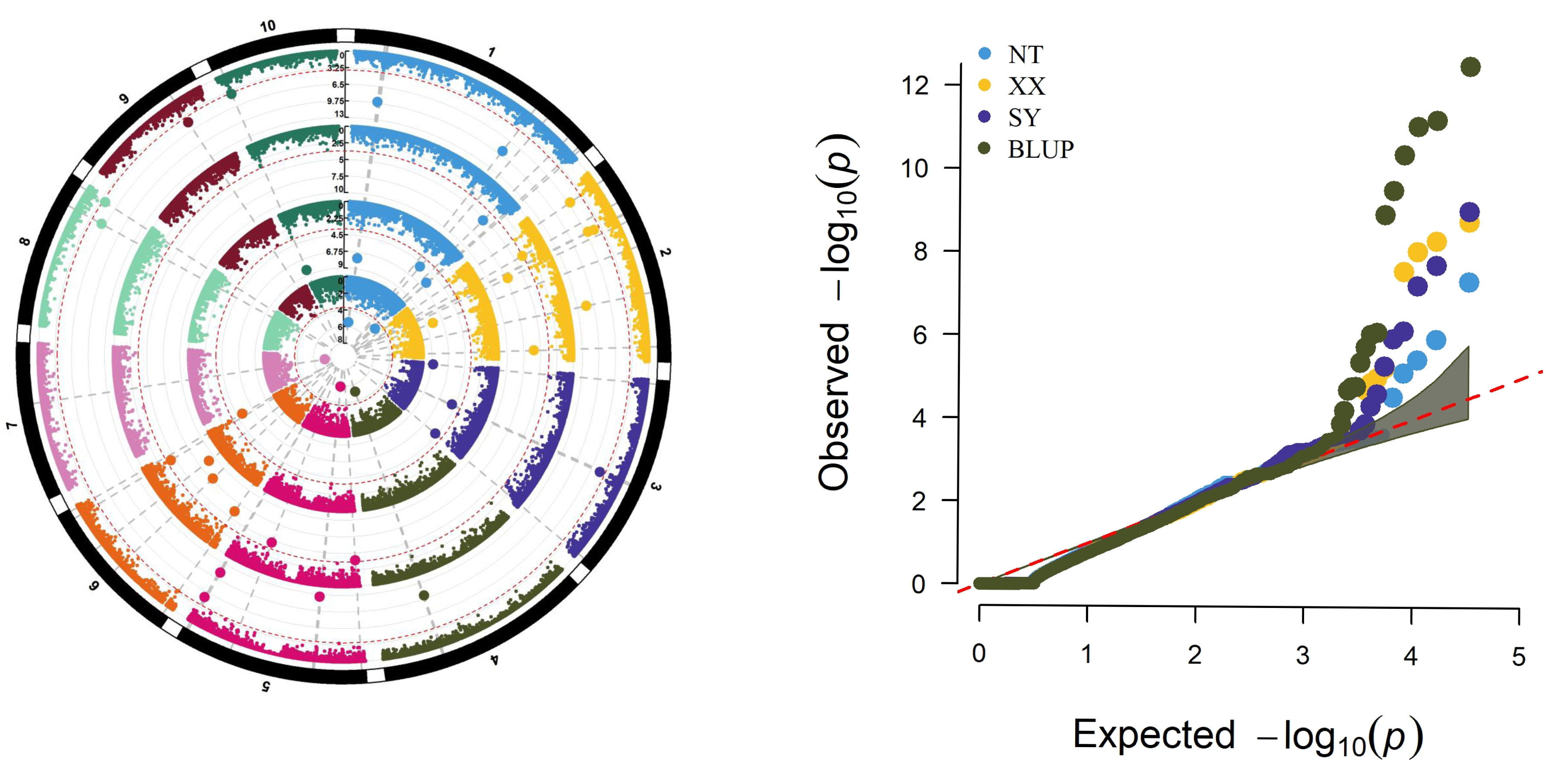

Supplement: Supplementary Figure 2 — Manhattan (left) and quartile-quartile (right) plots of ML-GWAS results using FASTmrMLM model. The four circles of Manhattan plot from inside to outside show the result in Nantong (NT), Xinxiang (XX), Sanya (SY), and best linear unbiased prediction (BLUP), respectively. [file Image_2.TIF]

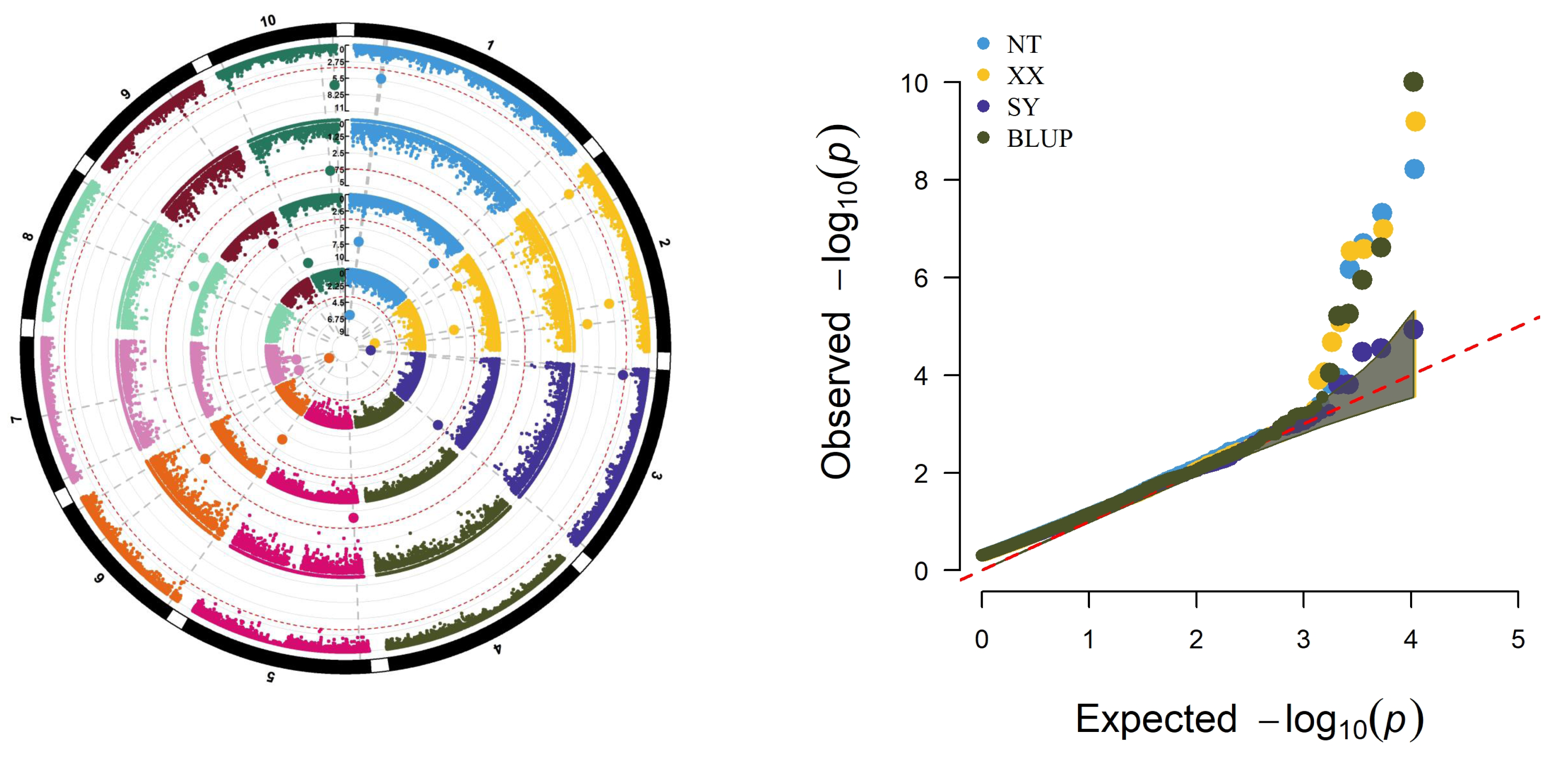

Supplement: Supplementary Figure 3 — Manhattan (left) and quartile-quartile (right) plots of ML-GWAS results using FASTmrEMMA model. The four circles of Manhattan plot from inside to outside show the result in Nantong (NT), Xinxiang (XX), Sanya (SY), and best linear unbiased prediction (BLUP), respectively. [file Image_3.TIF]

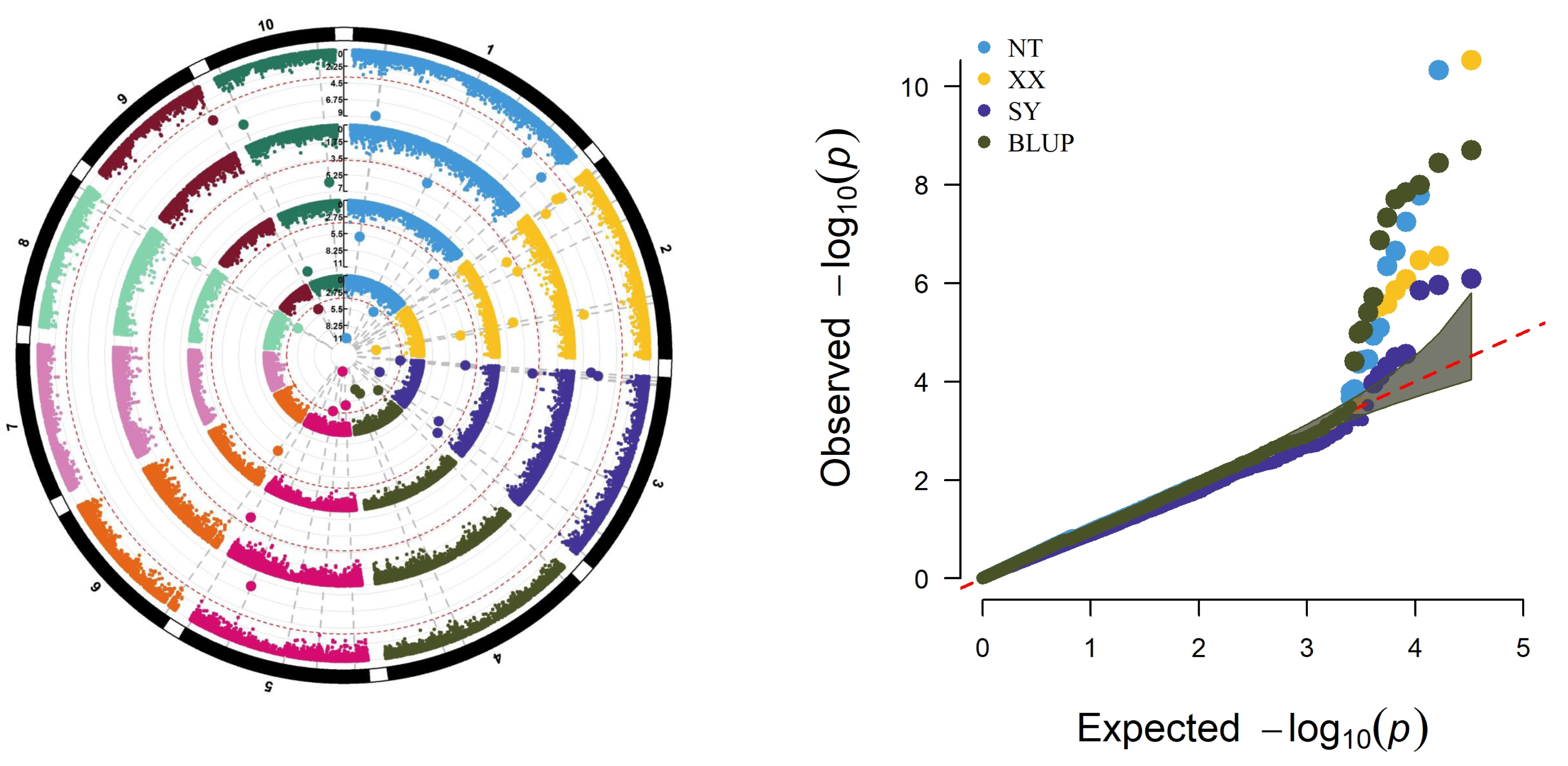

Supplement: Supplementary Figure 4 — Manhattan (left) and quartile-quartile (right) plots of ML-GWAS results using PKWmEB model. The four circles of Manhattan plot from inside to outside show the result in Nantong (NT), Xinxiang (XX), Sanya (SY), and best linear unbiased prediction (BLUP), respectively. [file Image_4.TIF]

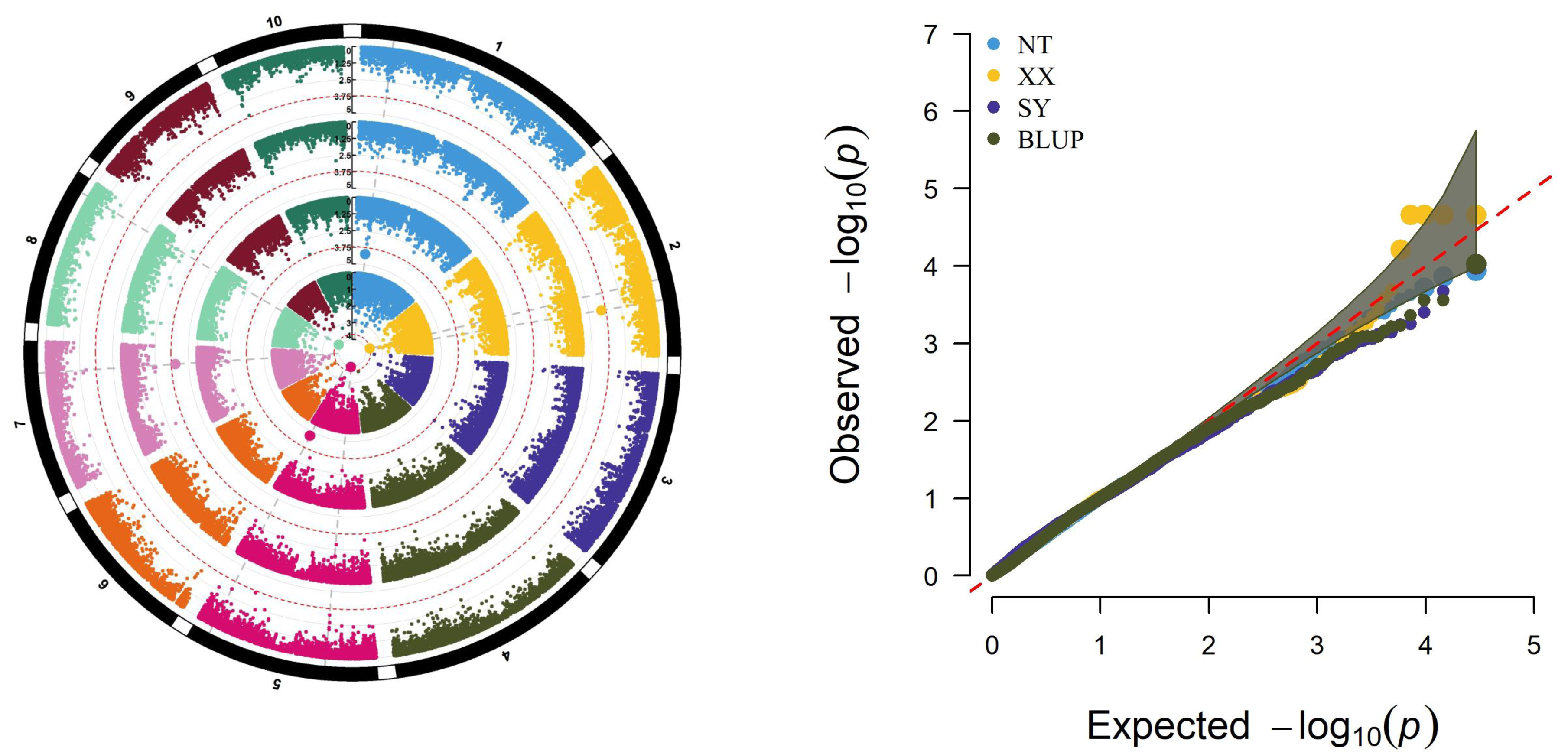

Supplement: Supplementary Figure 5 — Manhattan (left) and quartile-quartile (right) plots of SL-GWAS results using MLM model. The four circles of Manhattan plot from inside to outside show the result in Nantong (NT), Xinxiang (XX), Sanya (SY), and best linear unbiased prediction (BLUP), respectively. [file Image_5.TIF]
